# Intraluminal migration of a spacer with small bowel obstruction: a case report of rare complication

**DOI:** 10.1186/1477-7819-10-30

**Published:** 2012-02-06

**Authors:** Takayuki Ogino, Mitsugu Sekimoto, Junichi Nishimura, Ichiro Takemasa, Tsunekazu Mizushima, Masataka Ikeda, Hirofumi Yamamoto, Yuichiro Doki, Masaki Mori

**Affiliations:** 1 Department of Gastroenterological Surgery, Osaka University Graduate School of Medicine, 2-2 Yamadaoka, Suita, Osaka 565-0871, Japan

**Keywords:** spacer migration, locally recurrent rectal cancer, small bowel obstruction

## Abstract

The spacer placement is a prevalent procedure to separate the surrounding normal tissues from locally recurrent rectal tumor before the application of radiotherapy. However, complications could occur due to the foreign nature of the spacer. This report describes a case of 60-year-old man who had undergone radiotherapy two years earlier for a recurrent rectal tumor and presented with small bowel obstruction. A spacer was used before radiotherapy. Radiological assessment and laparotomy revealed the presence of the spacer inside the small bowel lumen. It is possible that the spacer established contact with the intestine, elicited local inflammatory reaction that facilitated the complete penetration of the intestinal wall without causing any clinical symptoms.

## Background

Radiotherapy is effective for locally recurrent rectal cancer. The risk of bowel radiation injury depends on the radiation dose. Basic radiation protection principles include increasing the distance between normal tissue and site of radiation, to reduce overall radiation exposure [1]. For this purpose, spacer placement between the small bowel and local recurrent rectal tumors before the application of radiotherapy is becoming a prevalent procedure. However, in a very few cases, radiotherapy can result in uncommon complications such as intraluminal migration of the spacer. Here, we present a case of complete spacer migration into the small bowel presenting as small bowel obstruction.

## Case presentation

**Figure 1:**
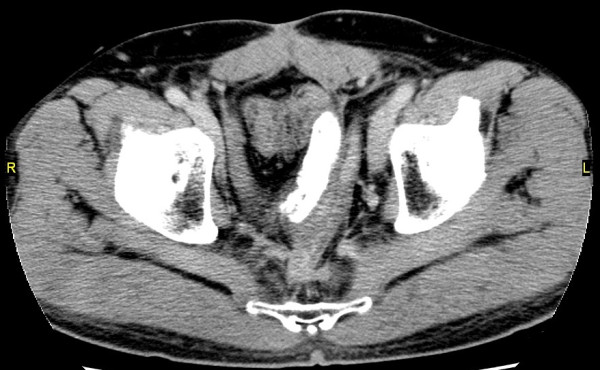
**CT scan showed spacer placed between the tumor and small bowel**.

The patient was a 60-year-old man with a negative past medical history. In June 2005, he was diagnosed with advanced rectal cancer with bladder invasion and underwent abdominoperineal resection with partial cystectomy and vesicoureteral anastomosis. The final TNM stage was stage IIIB (T4N3M0). Two years after the first surgery, follow-up positron emission tomography/computed tomography (PET-CT) scan showed locally recurrent rectal cancer in front of the sacrum. He opted to receive radiotherapy, but this proved difficult due to the close proximity of the recurrent tumor to the small bowel in the pelvic cavity. To prevent exposure of the small bowel to the irradiation, a polytetrafluoroethylene spacer measuring 15 × 10 cm was placed between the tumor and small bowel. The spacer was secured in place with 3-0 Vicryl suture. A CT scan showed the spacer was positioned appropriately in the desired location (Figure 1). One month after the second surgery, radiotherapy was performed without any adverse events. Systematic chemotherapy was continued and there were no sign of tumor growth.

**Figure 2:**
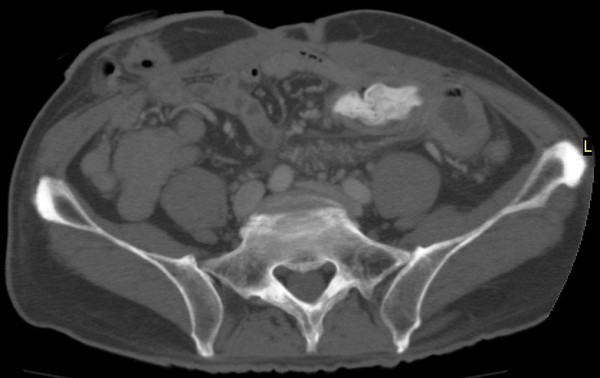
**CT scan showed spacer migration into the abdominal cavity**.

**Figure 3:**
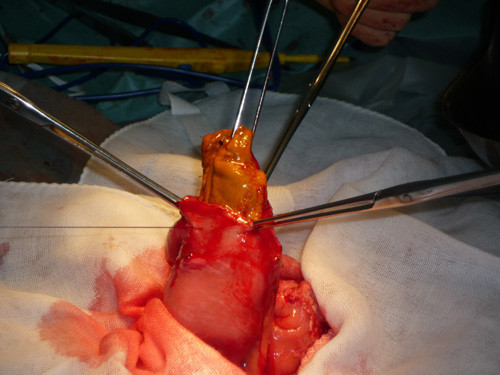
**Enterotomy showed the oval-like spacer had completely migrated into the lumen of the small bowel**.

Two years after the second surgery, he presented to the emergency department with left abdominal pain, nausea and vomiting. A CT scan of the abdomen showed dilated small bowel and spacer migration to the abdominal cavity (Figure 2). He was transferred to the operating room and underwent exploratory laparotomy. Exploration of the abdominal and pelvic cavities showed dense adhesions in the pelvic cavity but the spacer could not be located. Careful exploration of the abdomen revealed that the spacer had completely migrated into the small bowel lumen. Enterotomy was performed and the oval-like spacer was removed (Figure 3). He had uneventful recovery after the third surgery, and no evidence of tumor growth was noted in the last follow-up visit in April 2011.

## Discussion

Although surgical resection is considered the most effective therapy for locally recurrent rectal cancer, surgical stress and sacral nerve damage accompanied by high sacral resection often yield dismal results [2]. The use of radiotherapy as an alternative modality has increased in recent years, especially for locally recurrent rectal cancer due to its high efficacy. In radiotherapy for locally recurrent rectal cancer, the small bowel is the major dose-limiting structure. Spacer placement to physically separate the small bowel from the recurrent tumor can reduce the bowel radiation dose. Spacer should be sutured securely to cover the entire area of tumor. Usually, artificial material, greater omentum or muscle is used as a spacer. In this case, a polytetrafluoroethylene sheet was placed between the tumor and small bowel. Complications related to the use of the spacer artificial material include organ injury, bleeding, foreign body reaction, infection, and fistula formation. Complete migration of the spacer into the lumen of the small intestine is extremely rare. To our knowledge, this report is the first case of complete spacer migration inside the small bowel.

Intraluminal migration of artificial materials has been described previously, such as the migration of mesh after hernioplasty [3-8]. Agrawal and Avill [3] reported finding intravesicular mesh following laparoscopic transabdominal preperitoneal inguinal hernia repair. Celik et al. [4] reported intraluminal mesh migration into the splenic flexure following transabdominal preperitoneal repair of inguinal hernia, which was subsequently removed by colonoscopy. Benedetti et al. [5] reported a patient who presented with rectal bleeding 2 years after inguinal hernia repair and was found to have sigmoid colon perforation with the plug partially within the colonic lumen. Lange and coworkers [6] reported a patient with sigmoid penetration following transabdominal preperitoneal repair of inguinal hernia. Goswamiet and colleagues [7] reported mesh migration into the cecum after transabdominal preperitoneal hernioplasty. Borchert et al. [8] reported a female patient who presented with small bowel obstruction 7 years after obturator hernia repair. The above reported cases involved small bowel obstruction after bowel adhesion to the mesh rather than transmural migration.

Two published case reports have described complete migration of artificial materials into the small bowel [9,10]. Majeski [9] reported intraluminal mesh migration in a woman who had undergone incisional hernia repair with wire mesh 40 years prior to presentation with small bowel obstruction. A piece of wire mesh was found to have broken off and was within the lumen of the small bowel. Steinhagen et al. [10] reported complete transmural mesh migration from the abdominal wall into the small bowel one year after repair of incisional hernia with polytetrafluoroethylene mesh.

While migration of artificial material is quite uncommon, it is possible for mesh to translocate into the hollow abdominal viscus, although the exact mechanism involved in this process is not clear at present. Robinson and Levin [11] suggested that the retained foreign body, e.g., surgical sponges, becomes encapsulated by loops of small bowel and by peritoneal reaction. Riaz et al. [12] hypothesized the edges of the mesh might cause damage to the surface of nearby organs and evoke an inflammatory response leading to erosion and migration. Celik et al. [4] argued that it occurs when part of the bowel is carelessly included in the fixation of the mesh. Growswami et al. [7] proposed that adhesions at hernioplasty can predispose the patient to further adhesion formation leading to migration.

The above proposed mechanisms do not provide a reasonable explanation for the present case. Our patient was in good nutritional status and was not on long-term steroid use. How did the spacer pass from the outside of the small bowel into the lumen? A huge defect in the intestinal wall is required for the spacer to migrate into the lumen. However, this patient never developed sepsis-like symptoms that would be expected during the course. Based on the presence of dense adhesions in the pelvic cavity, we propose that the contact between the spacer and intestinal wall elicited local inflammatory reaction without systemic symptoms, during which the spacer eroded the bowel and ultimately penetrated the entire wall. However, the small intestine site containing the spacer was not near the pelvic cavity. This suggests that the spacer traveled within the lumen from the penetration site retrogradely to the site of small bowel obstruction. This case presents extremely uncommon complication of surgery using spacer. Such complication could be perhaps avoided by leaving non-viscus tissue between the spacer and intestinal wall, leaving no direct contact between the two.

## Conclusions

We experienced a very rare complication of intraluminal spacer migration with small bowel obstruction.

## Consent

Written informed consent was obtained from the patient for publication of this report and any accompanying images. A copy of the written consent is available for review by the Editor-in-Chief of this journal.

## Conflict of interest statement

The authors declare that they have no competing interests.

## Authors' contributions

TO wrote the main manuscript and MS performed the operation, revised the manuscript for important intellectual content, and gave the final approval for the version to be submitted for publication. All authors read and approve the final manuscript.
